# Metals: Fresh Track on Indoor Dust

**DOI:** 10.1289/ehp.116-a198

**Published:** 2008-05

**Authors:** Bob Weinhold

Dust is seldom innocuous. At the very least, it can earn homeowners a reputation for lax housekeeping. More important from a health perspective, it can also exacerbate asthma and allergies. Growing evidence suggests this humble material may pose other health threats caused in part by metals and other potentially toxic substances that hitchhike on core particles.

In traditional risk assessment, one common assumption has been that contaminants in indoor dust are just a dilute version of what’s found in the soil outside the building. New evidence from a team of Canadian researchers reveals just the opposite for many metals: indoor dust can be burdened by much higher quantities than in soils outside, and the indoor version can also be more orally bioaccessible (made available to the body through the digestive process). Based on her team’s findings, Pat Rasmussen, a research scientist with Health Canada, says the old assumption “is wrong, and can result in a serious underestimate of indoor exposures in residential risk assessments.”

This is one of the early results from the Canadian House Dust Study, scheduled to run from 2007 through 2010, for which Rasmussen is principal investigator. Besides metals, bacteria have been studied in homes in three cities; pesticides, alkylphenols, artificial musks, brominated flame retardants, organotins, parabens, perfluorinated compounds, phthalates, and triclosan also may be studied.

Based on samples from about 1,000 homes in 13 representative cities, the study is expected to set the first scientific baseline for various contaminants in urban Canadian homes. Once all study results are available, Health Canada and the Public Health Agency of Canada, two of the study sponsors, are expected to make recommendations on how best to deal with household dust and its associated contaminants.

For the pilot phase of the metals study, which was published in volume 14, issue 2 (2008) of *Human and Ecological Risk Assessment*, Rasmussen and her colleagues determined the copper, nickel, and zinc contents of archived dust and soil samples from 22 homes in one city. They found not only that the concentration of each metal was far higher in indoor dust than in outdoor soil (9, 3, and 7 times, respectively), but that each metal was much more orally bioaccessible in dust than in soil (13, 8, and 23 times, respectively). In addition, they discovered large differences in the organic and inorganic carbon contents, which were 5 and 10 times higher, respectively, in indoor dust than in soil. Through the use of synchrotron X-ray absorption spectroscopy, the team found that copper was more often bound with organic portions of indoor dust (e.g., sulfides such as cysteine, and possibly acetate and oxalate), while zinc was more often bound with the mineral portions.

“The X-ray analysis is rather novel, and the results informative,” says Andrew Turner, an associate professor of environmental sciences at the University of Plymouth, United Kingdom. He is not involved with any segment of the study and says he’s unaware of any other study of similar scope.

The team hopes its future studies using the synchrotron and novel applications of technology such as Elementar devices, specialized analytical instruments for examining nonmetallic elements, will help it pin down the specific sources of the indoor metals, whose concentrations tend to vary substantially from house to house in the same neighborhood and even from room to room in the same house for reasons unknown.

Results from earlier studies indicate that many other metals—including lead, cadmium, arsenic, mercury, aluminum, chromium, iron, manganese, and tin—are usually present in indoor dust. Rasmussen is studying the presence in dust of these other elements and is finalizing results for publication.

The mere presence of these metals does not necessarily pose a clear health risk, says Paul Lioy, associate director of the Environmental and Occupational Health Sciences Institute at the University of Medicine & Dentistry of New Jersey. But he says this study, which he did not participate in, adds several nuggets of new information, and he notes there are many indications that this general line of inquiry could prove very important: “Dust is an incredible marker of past exposures to persistent chemicals.”

Until more is known about the health effects of house dust, Rasmussen says the best approach for homeowners may be to play it safe and keep dust levels down by vacuuming (a HEPA filter is a good idea but not required, says Rasmussen), damp-wiping surfaces, and removing shoes when entering a house. For situations where the dust is known to be contaminated by a proven toxic source, such as lead paint in older buildings or mercury from a broken compact fluorescent lamp, more specialized cleanup methods are needed.

## Figures and Tables

**Figure:**
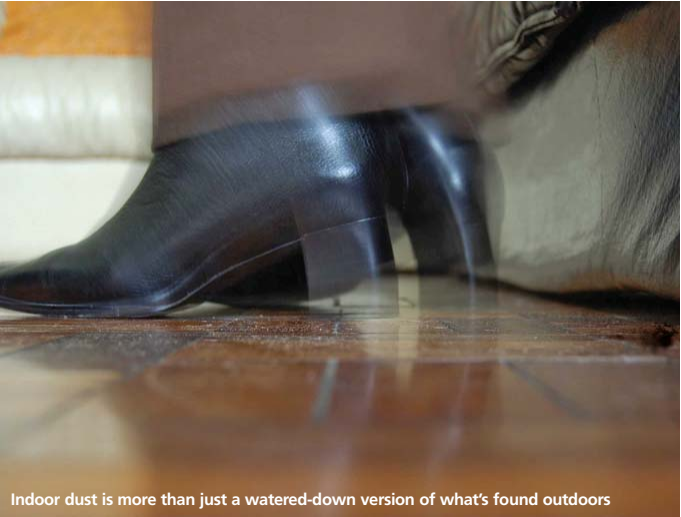
Indoor dust is more than just a watered-down version of what’s found outdoors

